# The global evolution of talent promotion within Olympic sports: A focus on the national systems and contribution of the former German Democratic Republic, Australia, and the United Kingdom

**DOI:** 10.3389/fspor.2022.1124234

**Published:** 2023-02-02

**Authors:** Juanita R. Weissensteiner

**Affiliations:** Pathways, Sector Performance, Policy and Planning, New South Wales Office of Sport, Sydney Olympic Park, NSW, Australia

**Keywords:** systems, policy, strategy, athlete, development, management, national policy approaches

## Abstract

In this chapter we chronicle and explore the global evolution of national level talent promotion through the lens and respective journeys of the former German Democratic Republic, Australia and the United Kingdom. Whilst ideologically vastly different, core elements of talent promotion were mirrored and extended within the next national iteration. Key learnings obtained from this historical and comparative exploration serve to provide excellent learnings for policy makers, strategists, practitioners and researchers to support the review and development of current and future national talent promotion systems.

## Introduction

The undisputed goal of nations within the “*global sporting arms race*” (1) is finding the right strategic approach to ensure sustainable high-performance outcomes on the world stage, including notably, at the Olympic Games. Central to achieving this objective is talent promotion – the effective recruitment, selection, development, and transition of pre-elite level athletes to a high-performance level.

**Figure 1:**
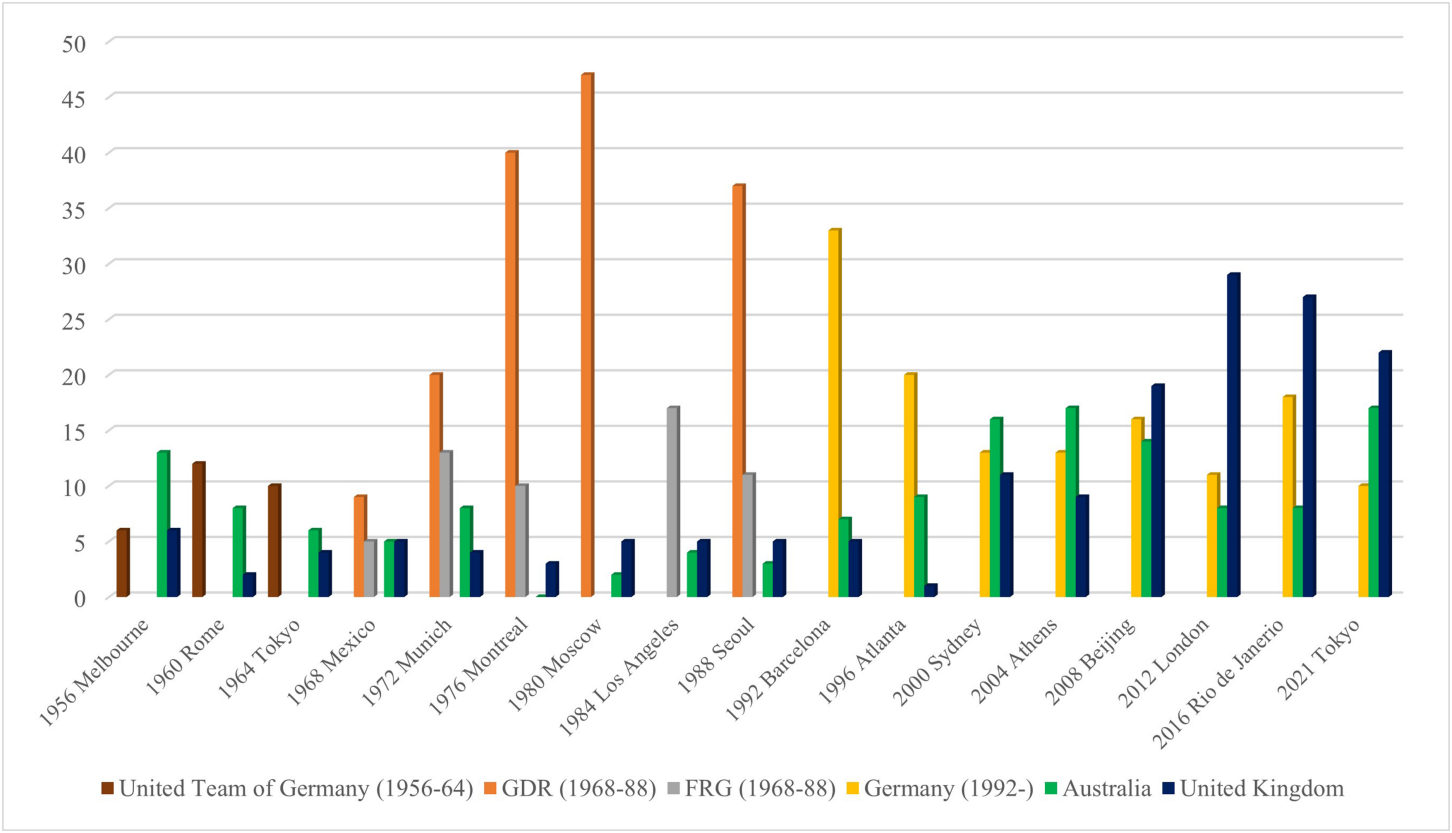
Olympic gold medal country tally from Melbourne (1956) to Tokyo (2021) Olympic games. Note: GDR and FRG were part of the United Team of Germany between 1956 and 1964. The FRG boycotted the 1980 Moscow Olympic Games, and the GDR boycotted the 1984 Los Angeles Olympic Games.

Recognised as one of the first national talent promotion systems established in the post-war era, the German Democratic Republic achieved rapid and significant Olympic success through the 1970s and 1980s. Albeit veiled by great secrecy and state censorship, the system was admired and emulated globally prior to its dissolution in 1989 and the exposure of its state-sponsored doping of athletes. Notwithstanding this fact, the system and many of its pillars, provided a legacy, directly influencing the build of talent promotion systems within emerging sporting nations such as Australia through the 1970s to 2000s and later, the United Kingdom, contributing to substantial Olympic success for both countries (see Figure 1).

Utilising a historical and comparative approach, in this chapter we will examine the chronology and contribution of these three national systems to the broader discipline of talent promotion within Olympic sports. Regardless of their obvious heterogeneity (i.e., ideology, culture, governance etc.) at the core of these systems were commonalities or “homogenous” aspects (i.e., policy, strategy, structures, delivery etc.) that were mirrored, adapted, and extended within the next system and enabled importantly, through the “transfer” of leadership, knowledge, and innovation. As Dennis and Crix (2) share, “*Such a legacy is not to be measured simply in what remains in place in Germany after unification … a legacy can take many forms. It might be ideational, structural or take the shape of an actual person, who brings with them ideas, conventions, technical knowledge, tricks of the trade and so on”* (p. 171).

The author is a former athlete and current policy maker, academic and practitioner, who has dedicated the last 20 years of their career to the evolution of talent promotion within Australia and internationally, including providing advice to the International Olympic Committee [see Bergeron et al. (3),]. Reflecting upon the evolution of these systems, provides excellent learnings and impetus for fellow policy makers and practitioners to guide their future planning and implementation.

## Former German Democratic Republic (1949–1990)

At the 1976 Montreal Olympics, the world first took notice of the German Democratic Republic, an emerging sporting superpower that doubled its gold medal haul at its home Olympics in Munich in 1972. As Dennis and Crix (2) share, “*Interest in finding out what made up the East German sports system rose sharply after the first international successes of its athletes*.” (p. 176). Within the following section, we will explore the genesis, elements and limitations of its national talent promotion system.

### Genesis and political context

Following the decimation of Germany after World War II, a key priority of the ruling Socialist Unity Party of Germany (*Sozialistische Einheitspartei Deutschlands* -SED) when it came to power in 1949, was the rebuilding of its entire national high-performance sports system (4–6). Prior to this time, high performance sport was amateur and diversified featuring autonomous sports clubs and associations and worker's sport (2).

Influenced heavily by the communist ideology and sporting success of its occupying force, the USSR (Union of Soviet Socialist Republics) (i.e., the USSR ranked second on the country medal tally at its first Olympics in Helsinki in 1952 and then first at the 1956 Melbourne Olympics), high performance sport became a political instrument of the authoritarian regime. Success on the world sporting stage, was a means of displaying to the world the physical prowess of its citizens, affirm the strength of its socialist ideology and values domestically, and confirm its superiority over its capitalist rivals including the Federal Republic of Germany (West Germany) (2, 5–8).

Specific to mass participation was the regime's “*sport for all*” policy. Walter Ulbricht, the first head of state of the German Democratic Republic, preached “*strength through physical culture and sport*” (7) and demanded that all citizens, young or old, participated in some form of physical activity and this was overseen by the State Committee for Physical Culture and Sport (*Staatliches Komitee für Körperkultur und Sport* - Stako). This was achieved though the prioritisation of physical activity and sport within its school network (including paramilitary disciplines such as close combat and grenade throwing), incentivised physical activity within people's workplaces and the hosting of local and national annual sports festivals (*Spartiakades*).

### National governance and priority

High performance sport was centrally governed through the centralist management and control of the SED's Central Committee Department for Sport and its related policy, planning and monitoring processes including a dedicated national operational plan for each sport, detailed performance targets and biennial monitoring (2). As Dennis and Crix (2) share, “…*the GDR was an authoritarian dictatorship and as such did not suffer the usual problems of interest mediation, lobbying and difficulty with policy implementation in liberal democracies. Given that sport policy was dealt with at the top of the hierarchy, decisions were made, policy was changed and implemented more swiftly and without the need for widespread consensus as is the case in democratic regimes*” (p. 181). Supporting the SED at a national level was the High-Performance Sports Committee (*Leistungssportkomission* - LSK), German National Olympic Committee (NOC) and the German Gymnastics and Sports Association (*Deutscher Turn- und Sportbund der Deutschen Demokratischen Republik* - DTSB) an “umbrella” organisation responsible for providing oversight of its Olympic National Sporting Federations (NSF) (2, 5, 8, 9).

The Stako also provided initial oversight of the German College for Physical Culture (*Deutsche Hochschule für Körperkultur -* DHfK), a national college established in 1950 dedicated to the education of physical education teachers and coaches, and the Research Institute for Physical Culture and Sports (*Forschungsinstitut für Körperkultur und Sport -* FKS), which oversaw research into high-performance sports. Both institutions were situated within the campus of Leipzig University and will be discussed later in this chapter. Other specialist national level institutions included the Research and Development Centre for Sport Equipment (*Forschungs und Entwicklungsstelle für Sportgeräte -*FES), which specialised in the development of sports equipment (e.g., boats, bicycles, skis, bob sleds, luges, speed skates, poles, etc.) and the National Sports-Medical Service (*Sportmedizinischer Dienst der* - SMD) which provided medical services and oversaw sports medical research, inclusive of performance enhancing pharmaceuticals, as will be discussed later.

Talent promotion was central to the SED's national high-performance policy and implementation. The core operational infrastructure of the regime's system was established between 1951 and 1956, including the build of state-of-the-art sporting facilities (e.g., elite training centres) across its territories through its *Golden* and later *Golden East* plans (Hallman et al., 2018), Children and Youth Sports schools (*Kinder und Jugendsportschulen* - KJS) and elite sport clubs such as SC Dynamo Berlin (2). Most sport clubs were state-sponsored and were under the strict control of the army, police, or the “Ministry for State Security” (Stasi - *Staatssicherheit*). It is reported that every tenth citizen of the regime were involved in the surveillance of their own family and friends, and it is alleged that within the sports system, it was more prevalent.

By the mid-1970s and 1980s, the majority of national level sport funding was allocated to the high-performance system including ongoing investment into prioritised “category A” Olympic sports (i.e., sports considered to have substantial medal prospects), its dedicated workforce of coaches and trainers and athletes within its *cadre* system. Ongoing clashes at a bureaucratic level ensued with the Stako insisting that more resources be assigned to support mass participation inclusive of children's and youth sport. However, as Dennis and Crix (2) share, mass sports participation “…*did not survive the voracious appetite for resources of elite sport*.” (*p*. 41) and “*sports for all*” was greatly underserved due to the regime's “*win at all costs*” mentality (2). As Dennis & Crix (2) continue, “*… there was no harmonious, iterative relationship between mass and elite sport: the latter was very clearly demarcated from the former. Over time, the East German authorities were unable to support all areas of sport and as such mass sport provision declined rapidly from the end of the 1970s until the state's collapse in 1989*” (p. 157–158).

### Systematic talent identification and development

Considering its relatively small populace (i.e., approximately 16 to 17 million citizens) and the increasing importance placed by the regime on achieving international success, the development of a national system to sift through, select and develop its sporting talent from a young age and enhance the talent pipelines of its prioritised Olympic sports, became an urgent priority of the SED.

**Figure 2:**
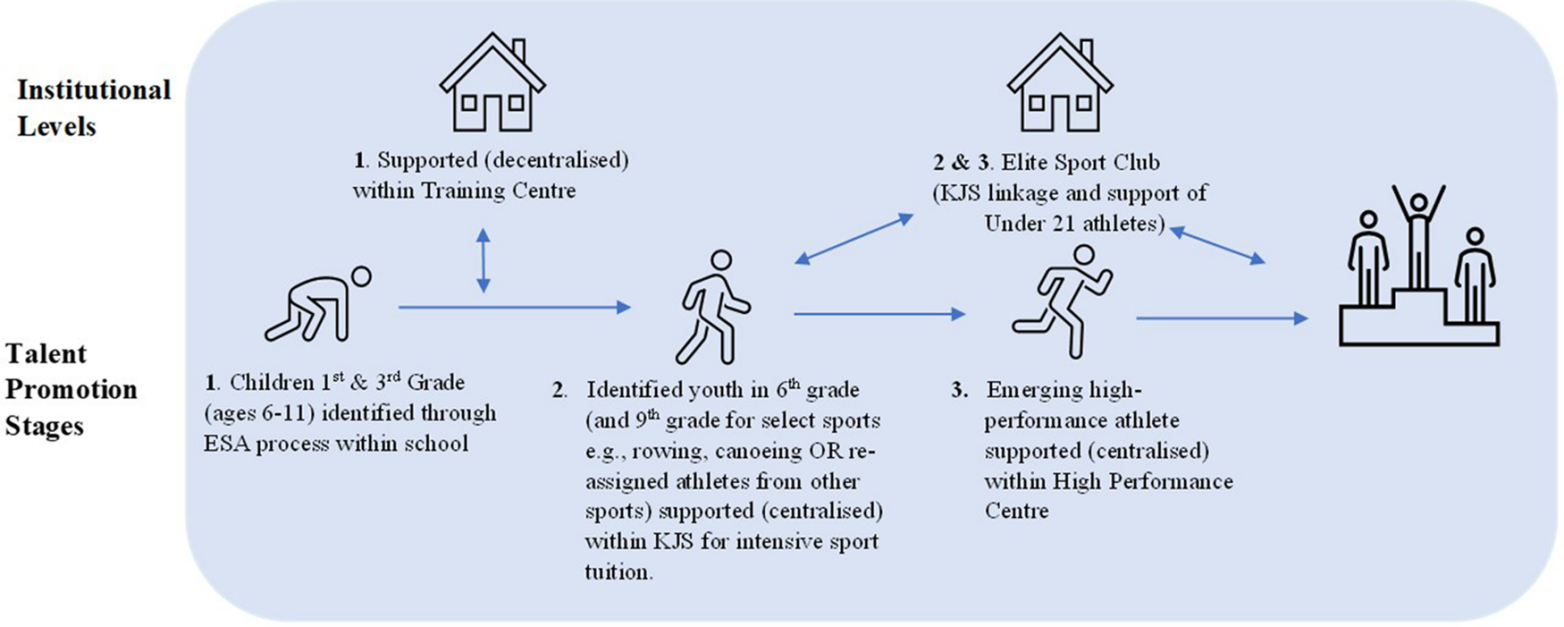
Schematic overview of the German Democratic Republic's three-step talent promotion process that existed from 1973 to 1989.

Central to achieving this outcome and depicted within Figure 2, was the establishment of a systematic and nation-wide process for identifying and developing young and prospective sporting talent which featured three progressive stages:

Stage 1 - 1st and 3rd grade school students were tested and selected for specific sports through a Uniform Inspection and Selection process (*Einheitliche* Sichtung *und Auswahl* -ESA) developed through the FKS and their early sporting development supported through decentralised training centres.

The ESA administered by physical education teachers throughout the school network operated nationally from 1973 until 1990 and resulted in the screening annually of over 200,000 children within grades 1 and 3 for specific sports (10). Prior to the ESA, youth selection relied on informal processes such as coaches eye assessments implemented by locally based trainers and schoolteachers.

Stage 2 - School students aged between 12 and 18 years (and school students aged between 6 and 8 years specific to artistic-composition sports such as gymnastics) were tested and selected into a KJS for intensive and centralised sporting development. The KJS concept was based on the USSR model of elite sport schools and featured an intensified sport curriculum and delivery, with the majority being boarding schools.

Following the 1968 Mexico Olympics, the KJS focussed solely on Olympic sports supporting approximately 10,000 athletes across the network. Each KJS were located strategically in regions with a good match of institutional and infrastructural conditions and were self-sufficient centralised talent promotion facilities, providing its student athlete population with intensive coaching and training support, access to catering and sports medical services. It is notable that the twenty-three KJS within the system—unlike all other schools in the country—were under the direction of the twenty-one sports clubs of the regime, not the educational authorities. Students were required to invest in six hours of sport and only two hours of academic tuition each day resulting in severe academic deficits of many athletes for the sake of more available time for sport.

Stage 3 – High-performance athletes received centralised support within one of eight dedicated National high-performance centres which were closely linked to and overseen by an elite sport club. Each of these centres featured state of the art and innovative training facilities and equipment such as treadmills, swimming flumes and hypoxic chambers and athletes had ready access to high-performance coaches, quality daily training environments and equipment, interdisciplinary service support and sports medicine and travel support for competition.

Athletes at this level were able to train fulltime without risking their amateur status for Olympic level competition. Athletes who were successful in being categorised within the *cadre* system received scaled athlete payments (i.e., dependent upon what level they were at) through a dedicated sports foundation and vocational opportunity and support including industry traineeships and jobs.

This systematic approach provided a very structured athlete pathway and eco-system where the delegation, responsibilities, collaboration, and alignment of key stakeholders underpinning an NSF and the role and contribution of talent promotion facilities, was well defined. The capacity of the system was substantial, supporting thousands of athletes annually within each stage (6).

Talent development within each stage was informed by “scientifically based training systems” developed by the FKS which included the “*Framework for Training Concepts*” - set prescriptions of age-related training volumes specific to each type of sport (i.e., aquatic, endurance, combat, strength and power, game sports and acrobatic) (4).

An athlete's development was longitudinally tracked inclusive of their holistic profile, chronological age (and later also biological maturation) and performance outcomes. An athlete's performance prognoses were represented on a 100-point-scale and interpreted in relation to their training age for their sport. In turn, this collective data capture and analyses, was intended by the FKS to inform the further refinement of athletic norms and benchmarks and confirm the prognostic capability of the broader talent promotion approach.

### Investment into coach development

Coaches delivering at every level of the system, were required to be university qualified (overseen by the DHfK) and formally accredited by their sport and commit to ongoing professional development. In return, coaches were employed on a full-time basis and renumerated by the state and received rewards (including badges) and bonuses for performance success. Graduate coaches were extremely knowledgeable, skilled, and adept in implementing a strong pedagogical approach to athlete development (including effective periodisation inspired by Russian physiologist Leo Matveyev and later Romanian Tudor Bompa) and comfortably worked side by side within the daily training environment with sport science and sport medicine practitioners to assist their planning and delivery and facilitate the interdisciplinary case management of their athletes.

### Learnings and reflections

With the dissolution of the regime and the subsequent release of archived documentation chronicling the system, the ethics and success of the system came under question, when the systematic doping of its athletes even at a young age, was uncovered (6, 7, 11, 12). As Barker (11) shares, “*Often the anabolic steroid ‘Oral Turinabol’ was administered in little blue pills. Athletes and swimmers were often told that these were ‘vitamins’. Sometimes they were forced to sign confidentiality agreements*.” Through the 1980s, the national government in their aggressive pursuit of records and medals no matter the cost, invested over five million German marks annually to investigate doping substances as part of its State Plan 14.25. At the time of reunification in 1990, the FKS was allegedly overseeing twenty-one research projects investigating the effects of different doping substances on athlete performance. The performance differential between genders was significant (i.e., the success of female athletes was seven times that of male athletes), reflecting strong sex-specific variation and impact of doping on performance.

The long-term health and wellbeing of many of its athletes were greatly impacted by long term steroid usage culminating in devastating and irreversible consequences including infertility, birth defects, advanced heart disease, liver failure and gynecomastia (breast growth in males). Consequently, several coaches, administrators, and physicians including the former head of the DTSB and President of the NOC, Manfred Ewald, and Chief Doctor and Vice-Director of the SMD, Manfred Höppner were subsequently convicted of “*intentional bodily harm to athletes and minors*” and served lengthy gaol terms for their involvement (11).

Closer academic scrutiny of the system revealed several other limitations including -

1. its low cost-benefit ratio, dividends, and efficiency [see Güllich et al. (13); Güllich & Emrich (4, 14)]. As Güllich et al. (13) share, “*The system was effective in terms of international medals. On the other hand, its ‘tons ideology’ was oriented at effectiveness rather than efficiency, and by the 1980s, it had developed extreme requirements of resources”* (p. 58). With the fall of the regime, most of the reported six thousand coaches developed through the system, did not secure further employment domestically or internationally.
2. the selection process, cost-benefit, and impact of the KJS (4, 15). Approximately half of the athlete cohort that represented the German Democratic Republic at the 1988 Seoul Olympics, had failed to meet the selection criteria for admission to a KJS but were recruited anyway, and a similar percentage had initially been selected for a different sport and were later “delegated” to their Olympic sport. Many youth athletes were required to relocate and live on site (board) and contend with very high sporting demands and expectation, enjoyed limited recreational time and autonomy, and had limited connection to their families and social peers, for support.
3. the scientific rationality and rigor of the ESA athlete selection process. The reported prognostic validity of the assessment battery has since been questioned subsequent to allegations that it was empirically falsified. Despite inclusion of estimates of biological maturation to moderate an athlete's results and hence their sport suitability, this only marginally improved the prognostic validity of the test battery which whilst it purported to be “multi-dimensional” was contingent on anthropometric and physical markers. Subsequently, early maturing youth were commonly matched with strength-based sports and late maturing youth, with coordinative-based sports (2). Additionally, errors in data collection were also common (2).
4. the high rates of reported churn (i.e., burn out and drop out of athletes) at each level. Only a very small percentage of athletes that started the journey in their youth, continued within the sport and achieved success at a senior level [see Vaeyans et al. (16)]. Compared to their West German competitors, athletes were characterised by an earlier age of recruitment and specialisation, limited levels of sport sampling, earlier competitive success, and higher intensities of training over shorter developmental timeframes (4, 13). There was a presumption within the system, that early selection and subsequent sport specialisation based on early (junior level) indicators of performance, afforded a longer developmental period and that large volumes of training equated to high performance success but instead it contributed to high injury incidence, athlete burnout and dropout (4, 13, 15).
5. ongoing pressure and high expectations that were placed on athletes and the subsequent impact on their psychological wellbeing and opportunities within the system. Being a communist dictatorship, the system was characteristically restrictive and oppressive. Athletes and their families who were not ideologically conformist were banned from the sport system and devoid of vocational opportunities including attending university. Athletes who did not fulfil performance expectations were not admitted to the university subject they wanted, nor admitted to university at all.

As Dennis and Crix (2) contend, “*It is interesting to note that if the GDR had not collapsed in 1989 and ceased to exist in 1990, the sports ‘miracle’ is likely to have run aground by its own accord. There is growing evidence of declining resources, declining numbers of children to ‘stoke’ the system to keep it functioning, growing popular resentment against the extravagance what was the elite sport system and growing evidence that the doping programme had reached its limitations*” (p. 196).

### Final comments

Notwithstanding its questionable ethics and efficiency, remnants of the former talent promotion system of the German Democratic Republic including its elite sport schools' network and investment into state-of-the-art research, technology and innovation continue today within the modern German sports system, many decades after its fall. As Dennis and Crix (2) contend, “… *doping was one of the basic ingredients of an already formidable set of integrated elite sport development structures*” (p. 177). At an international level, its systematic approach to talent promotion, served as a legacy influencing the “build” of subsequent national systems through the 1970s and 1980s including Australia. The relative influence and contribution of the system and the subsequent evolution of talent promotion within Australia, will now be discussed.

## Australia

The advance in professionalism and subsequent international level success of the German Democratic Republic as well as of China, the USSR and Eastern Bloc countries, caught the attention of Australian bureaucrats, sport administrators and scientists in the early to late 1970s who were at the helm of formulating the blueprint for the modern Australian sporting system. A contributing factor to the collective-level success of the German Democratic Republic, the state-sponsored doping of its athletes, was unbeknownst to these keen observers but would be revealed later, after its fall in 1989.

The Australian high-performance system mirrored several of the key pillars of the former regime albeit not in its entire complexity, nor implemented in such a closed, authoritarian, and ethically questionable way (refer to Table 1 for a summary). For a country with a relatively small populace [i.e., currently just over 26 million citizens and ranked 52nd highest globally according to the website Population Australia (17)], “*punching above its weight*” on the international sporting stage, has long been synonymous with Australia's national identity and culture since its colonisation. Australia based on per capita of population, is considered one of the most successful national high-performance systems in the modern era of Olympic sport (i.e., for every 832,000 of its citizens, Australia achieves an Olympic medal) (18).

**Table 1:** Similarities and differences between the national talent promotion systems of the former German Democratic Republic and Australia.

Similarities	Dedicated federal policy and funding specific to high-performance
	Establishment of a National Institute of Sport and subsequent satellite network of state and territory high-performance institutes and academies
	Substantial federal investment into the build of state-of-the-art infrastructure and facilities
	Targeted federal level investment into prioritised Olympic sports
	Systematic athlete profiling and talent identification and development
	Provision of centralised daily training environments inclusive of quality coaching, sport science/medicine service provision, testing and individualised planning for athletes
	Scholarships and direct funding to athletes
	Investment into coach education, professional development and accreditation
	Investment into sport science/sport medicine research and innovation and integrated service provision for athletes
Points of difference	Lack of authoritarian and rigid governance, organisational and personal implications for poor Olympic performance
	Talent pathways and promotion not central to federal high-performance policy, implementation, and evaluation
	Nil state-sponsored implementation of athlete doping
	Diversified and complex talent pathways
	Coaching workforce not directly renumerated by the state
	Smaller cohort of sports schools but not central to national talent promotion strategy
	Non-professional club system which is not state sponsored

Today, Australia's high-performance system comprises a network of formally recognised National Sporting Organisations that are funded and supported by the federal agencies of sport including the national Office of Sport, the Australian Sports Commission (ASC), the newly formed Sport Integrity Australia, the Australian Institute of Sport, Australian Olympic Committee, Paralympics Australia, and the Australian Commonwealth Games Association. National Sporting Organisations (NSOs) are supported by a network of State Sporting Organisations (SSOs) funded and supported by their respective state or territory government departments, who in turn have oversight of a state institute or academy, regional level sport associations and academies, local amateur clubs and schools including public, catholic and independent schools and a small number of state sports schools (e.g., NSW Sports High Schools Association established in 2014 includes seven sports high schools, six of which are situated in the city of Sydney). This complex and diverse sports system as will be discussed later, provides an ongoing challenge with respect to the effective choreography and governance of talent promotion within Australia.

In this section we will chronicle the evolution and adaptation of Australia's national talent promotion system specific to Olympic sports.

### Genesis of Australia's high-performance system

Being a liberal democracy, Australia has enjoyed an open and diversified society and since 1901, possesses a federated system of national government, inclusive of six states and two territories, each with their own heads of government and underpinning network of metropolitan, regional, and remote local government areas and councils.

In post-war Australia, high performance sport was amateur, with minimal financial assistance at a state and federal level of government (19). Pockets of organic talent promotion through small club and coach-led programs had proven successful. At its first home Olympics in Melbourne in 1956, Australia placed second on the medal tally and gold medal winning athletes Betty Cuthbert, Shirley Strickland and Dawn Fraser became “*Aussie*” Olympic legends. Athletics coach, Percy Cerutty who's self-developed and unconventional “*Stotan*” training program embracing a holistic regime of natural diets, mental stimulation and resistance training to exhaustion within the sand dunes near his Portsea base in Victoria, was incredibly effective, nurturing a squad of world-class middle-distance runners including Betty Cuthbert but also Olympic champion and world record holder Herb Elliott and Olympic bronze medallist John Landy (20).

By the early 1970s however, Australia's amateur approach was quickly falling behind and unable to keep pace with the dedicated national systems and professional approach of the USSR, the German Democratic Republic and its Eastern Bloc allies, including Romania and Hungary (Bloomfield, 2003).

In direct response, the late John Bloomfield recognised as the chief architect of the modern Australian sports system and a longstanding and respected advocate, was commissioned in 1973 by the then Labor government and Australia's first federal sports minister Frank Stewart, to prepare a report titled “*The role, scope and development of recreation in Australia*” based on his keen observations and critique of international systems. Bloomfield's key recommendations included the establishment of a national sporting institute envisioned as a national centre of excellence, structures, and processes specific to effective athlete identification and development and federal investment into coaching and sports science/sports medicine disciplines. Bloomfield was also adamant that grass roots programs be established within the school and community sport network to facilitate the physical activity and fitness of youth. These collective recommendations were in accordance with then Prime Minister Gough Whitlam's view of sport as a vehicle for improving the overall welfare of the nation and “*a legitimate focus for public policy*” (21). However, due to a change in government in 1975, this plan was not realised immediately. Allan Coles in 1975 was commissioned by the subsequent Liberal government led by Prime Minister Malcolm Fraser to chair the development of the “*Report of the Australian Sports Institute Study Group*”.

At the subsequent Olympic games in Montreal, Canada in 1976, Australia failed to win a gold medal, placing 32nd on the medal tally. At the following Commonwealth Games in 1978 in Edmonton, Canada, Australia finished third behind the host nation and England. These collective poor performances “*thrust sport into the glare of the political spotlight*” [see Nihill & Drane (22) p. 13] and provided the urgency and catalyst for change commencing with substantial federal investment into Australia's high-performance system (21). The late Bob Ellicott, a minister within Malcolm Fraser's Liberal government decreed in 1980 with strong bipartisan support, that the Australian Institute of Sport (AIS) be established in Australia's capital city of Canberra, considered to be “*an investment in the nation and…. the future of Australian sport*” (21).

The AIS opened its doors soon after in 1981 and state of the art facilities were built on the campus including the National Indoor Sports Centre, a track and field stadium and later, a tennis hall, swimming centre and gymnastics centre. Soon after a dedicated sports science/medicine centre, administration building, national sport information centre (the first of its kind in the world) and residential complex were completed. Soon after, the AIS for a time, also served as a national training centre for non-residential sports such as Indoor Volleyball (the author was a joint Australian Volleyball/AIS scholarship holder in 1986, relocating to Canberra from Port Macquarie in regional New South Wales).

In 1985, the Australian Sports Commission was established, its role being to “…*fulfil the role of a coordinating body for sport—to foster cooperation, to allow for greater involvement of sports bodies in decision-making about sport and to broaden the financial base for sport*” [Jolly (21) p. 10].

**Figure 3:**
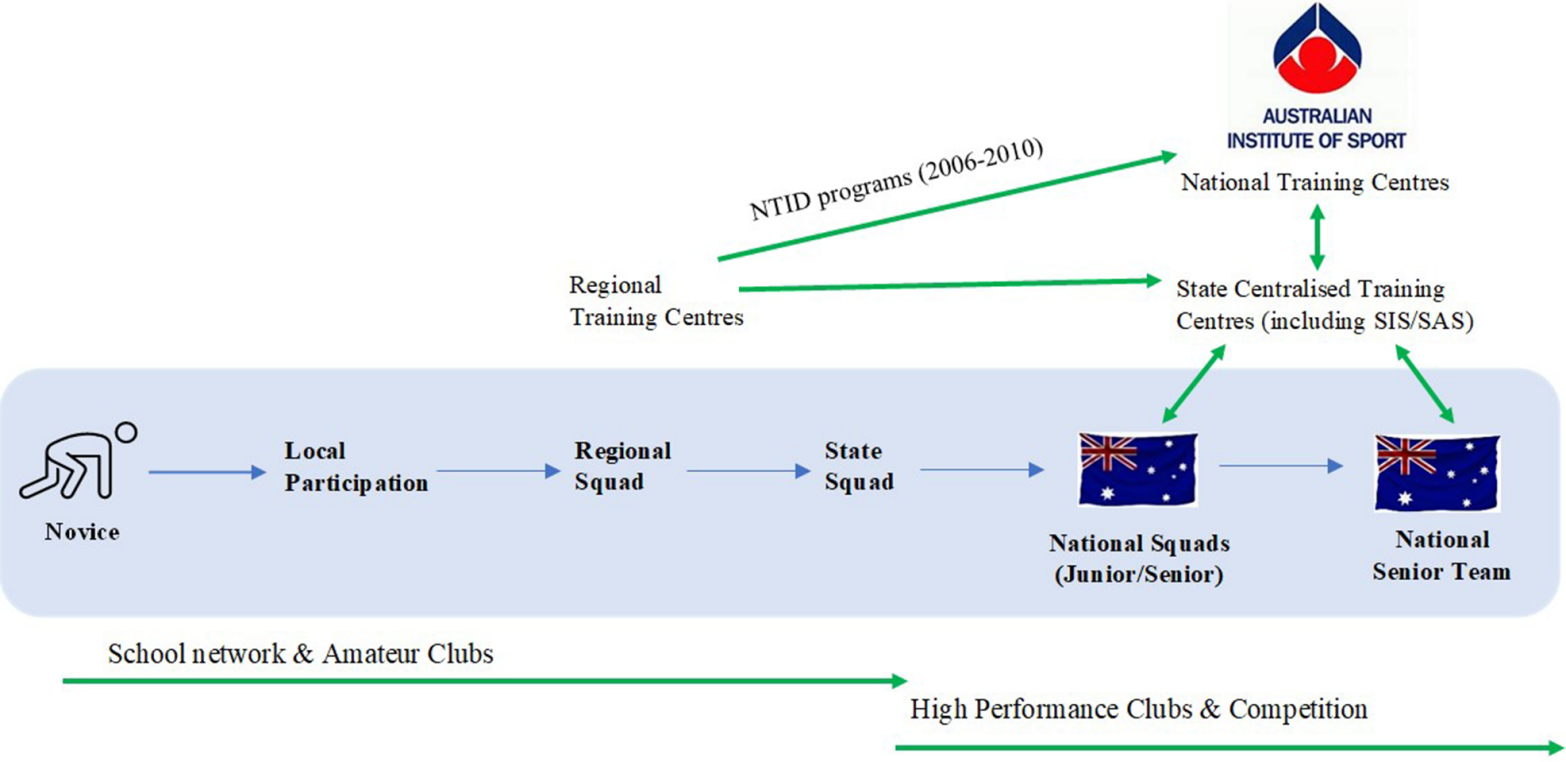
Talent promotion pathways within Australia pre-2014.

The initial intake of AIS athletes comprised of one hundred and fifty-three athletes across eight sports, basketball, gymnastics, netball, soccer, swimming, tennis, athletics, and weightlifting. At its peak, the AIS managed thirty-five separate programs within twenty-six sports, and the typical makeup of squads, were a mix of mature international-level performers and promising, emerging athletes. For these emerging athletes, particularly the many that originated from regional and rural Australia (23) the AIS offered a well-resourced and supportive centralised high-performance daily training environment and critical “steppingstone” to national representation (see Figure 3).

Each scholarship provided residential accommodation (initially a few short kilometres from the campus, but later within the AIS campus), high quality coaching, access to state-of-the-art training and competition facilities and equipment, interdisciplinary sport science-medicine service support (including education, individualised testing, planning, and monitoring), uniforms, meals, academic tutoring, travel, and domestic and international competition. For younger athletes in residence such as gymnasts, house parents, supervisors and mentors were assigned to chaperone and support. A requirement of each AIS scholarship was that athletes were expected to commit to concurrent educational or vocational training or work part-time. A dedicated sports studies faculty offering sport science/coaching, administration, and journalism, was established at the nearby Canberra College of Advanced Education (CCAE now known as the University of Canberra) under the leadership of renowned academic and sport scientist, the late Frank Pyke. Many scholarship athletes and their coaches within the AIS sports programs undertook these courses. There was strong linkage between these courses with practicums routinely taking place within the AIS environment and connection with its practitioners. Many graduate coaches, sport scientists and administrators from this college (the author being one of them), progressed to working within the AIS, the broader Australian system and internationally.

After the announcement in 1993 by the IOC that Australia had secured the hosting of the 2000 Olympics in Sydney, the federal government substantially increased its high-performance funding through its Olympic Athlete Program (OAP) which was administered by the AIS. Through the OAP, athletes were provided direct financial assistance, and were able to access more interdisciplinary sports science/medicine service support, professional coaching and dedicated career and educational support. The National Athlete Career and Education (ACE) program (later adapted into Personal Excellence and currently known as Athlete Wellbeing and Engagement) initially developed at the Victorian Institute of Sport, was delivered throughout the AIS and SIS/SAS network from the mid-1990s and was a world-first initiative dedicated to supporting an athlete's educational and vocational training and sport-life balance (24) and was later emulated by the United Kingdom.

Supporting an athlete's development (and importantly their coach), was a world-class and integrated sport science/sports medicine workforce within the AIS. The founding departments of the AIS featured expert and passionate scientists and practitioners and comprised of physiology, sports medicine, sport psychology, physical therapies and biomechanics. Later the disciplines of sports nutrition, performance analysis and skill acquisition were added. This vibrant eco-system embraced an unwavering culture of excellence, working collaboratively to provide servicing to its athletes, guide and support its coaches, and lead innovative research, providing an ongoing legacy for these disciplines across the national high-performance network and internationally for many decades to come. For instance, its Physiology department led the implementation of Australia's first talent identification program and later the Talent Search program (both of which will be discussed a little later), developed cooling jacket technology to support recovery, a detection test for erythropoietin stimulating agents, altitude adaptation (including a custom-built altitude house) and wearable micro technologies providing real time monitoring and feedback to athletes and their coaches. Internationally acclaimed sports nutritionist Louise Burke established its Sport Nutrition department, one of the first in the world, which has since been emulated across the globe.

The AIS's inaugural director was internationally renowned swimming coach, the late Don Talbot who had coached more than thirty Olympic and world record swimmers internationally (Canada and the United States of America) and within Australia including Olympic swimming twins, Ilsa and John Konrads (25). Drawing upon his personal learnings from working within the United States and Canadian systems, Don oversaw the prioritisation of quality coaching within the AIS including the provision of “apprenticeship” positions. Coaches were renumerated, benefitted from ongoing professional development, networking and learning from other AIS coaches, and were supported within the daily training environment by sports science/sports medicine practitioners (25).

The AIS also drew the attention of many international coaches including from Romania (e.g., Reinhold Batschi, inaugural AIS Rowing director) and the former German Democratic Republic. Internationally renowned cycling coach, the late Heiko Salzwedel who was both a cyclist and coach developed through the former system, became the inaugural head coach of the AIS's road cycling program from 1991 to 1998 (26, 27). Heiko like his East German coaching compatriots, brought with him a strong pedagogical and professional approach to coaching characterised by meticulous athlete planning and periodization.

Between 1982 and 1996, a satellite network of State and Territory Sporting Institutes and Academies (SIS/SAS) were established to support the decentralisation of some AIS programs including hockey (Perth, Western Australia), cycling (Adelaide, South Australia) and squash, canoeing and diving (Brisbane, Queensland).

The contribution of the AIS and SIS/SAS in supporting the effective talent promotion and subsequent international success of many of Australia's finest Olympic athletes, cannot be understated. For example, at the Sydney 2000 Olympics, the majority of athletes representing Australia were or had been supported through either an AIS, SIS/SAS, or co-badged scholarships (28). As Nihill and Drane (22) share, “*It (the AIS) took hold of the undeniable talent of Australian athletes, witnessed many times before, and applied a structured, supportive, and professional approach to the ongoing development of sport. It introduced Australians to professionalism in sport. It recruited coaches. It built infrastructure. It embraced sport science – Australian style, not Eastern Bloc. It exposed elite athletes to international competition. And it nurtured talent, opening up pathways for young elite athletes through a scholarship system designed to make them not just better athletes but better Australian citizens in life after competition*.” (p. 10).

### Transformative period of talent identification and development

To support Australia's sustainable Olympic success, it was imperative that innovative approaches to “flush” the pipelines of Olympic sports (including AIS and SIS/SAS scholarship programs) with prospective talent, became a key focus of the early AIS to lead on behalf of the national sport system. Prior to this time, the predominant approach was through talent selection from within a sport (29).

Following in the footsteps of Bloomfield and colleagues who implemented a scientific approach to talent identification in swimming in the early 1970s and 1980s inspired by those adopted within the German Democratic Republic and Eastern Bloc [see Bloomfield & Blanksby (30) and Bloomfield (31)], Allan Hahn, AIS physiologist and coach Peter Shakespear, drew inspiration and insights from the then Romanian women's rowing program, which won five gold medals at both the 1980 Moscow and 1984 Los Angeles Olympics (32–34). Allan and Peter established alongside AIS colleagues including the late Doug Tumilty, Australia's first talent detection program in 1987 in the sport of rowing. The initiative was fundamentally a “*proof of concept*” project – an opportunity to apply a scientific and detection approach to talent identification (i.e., source youth athletes from outside the sport) like Romania and the German Democratic Republic and confirm its viability within the Australian context [see Gulbin (29) and Hahn (32)].

In addition to supplementing the talent pipelines of rowing within Australia with numerous athletes gaining full AIS scholarships and achieving national representation at World Championship level between 1989 and 2004, the pinnacle achievement of the program was the successful pairing of Megan Still (now Marcks) and established rower Kate Slatter (now Allen) who became the first female crew to win gold for Australia at the 1996 Atlanta Olympics. The achievements of this initiative were not limited to the athletes it unearthed but also the coaching expertise it nurtured. Paul Thompson, the coach of Megan Still and Kate Slatter and a former elite rower himself, went onto achieve further world-class success within the United Kingdom system.

The success of the AIS rowing initiative within a relatively short time frame, fuelled great interest from other sports and led to the establishment of a similar initiative within the South Australian Sports Institute (SASI) in 1993 in partnership with Cycling Australia. Like the AIS rowing initiative, this program achieved international success quickly with podium success at the 1996 Junior World Championships, gold at the 1998 Kuala Lumpur Commonwealth Games and two top ten finishes at the 2000 Sydney Olympics.

**Figure 4:**
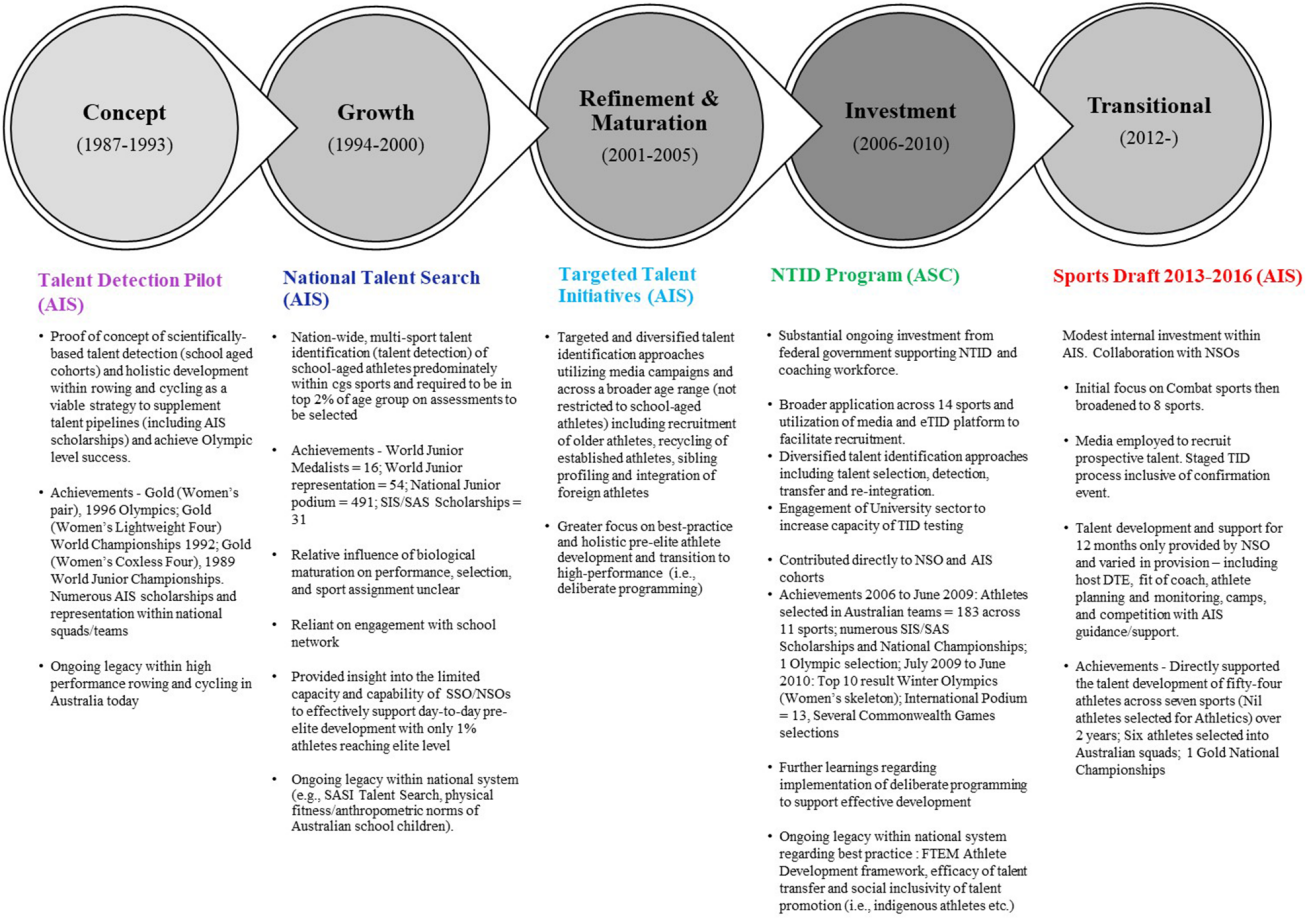
Evolution of talent identification and development under the leadership of the AIS (1987 to 2016). Contributing References: Abbott (35); Australian Institute of Sport (36); Australian Sports Commission (37–40); Ferguson (24); Gulbin (29); Gulbin et al. (41); Hahn (32, 33); Hoare (42, 43); Tomkinson et al. (44).

These “concept” initiatives were pivotal for Australian Olympic sport, signalling the start of a *transformative* evolution of innovative talent identification and development led by the AIS over the next two to three decades. The progressive phases constituting this transformative period are well described by Gulbin (29) as “*concept*”, “*growth*”, “*refinement and maturation*”, and “*investment*”. This fruitful period included the innovative computer-based “sport counselling” program *Sport Search* and subsequent National *Talent Search* program led by Deborah Hoare (now Latouf) which recruited school-aged children through talent detection and relied on state and national sporting organisations to manage an athlete's daily training environment, and the *National Talent Identification and Development* (NTID) program led by Jason Gulbin and informed by the learnings from *Talent Search* that incorporated diversified approaches for talent identification (i.e., selection, detection, transfer and re-integration of older aged established athletes) and expanded in capacity and capability through partnership with over 40 Universities and an electronic recruitment platform (eTID) and dedicated development programs on behalf of fourteen Olympic sports overseen by a workforce of NTID practitioners and coaches. Adapted from Gulbin (29), we provide in Figure 4 an overview this transformative period in talent identification and development, led by the AIS.

It is noteworthy, that these initiatives operated concurrent to and may have benefitted from, the implementation of the internationally recognised *Aussie Sports* program, delivered by the ASC as part of its *Active Australia* strategy between 1986 and 2003 (24, 45). *Aussie Sports* not only empowered the physical literacy and positive sport development of children and youth through participation within modified sports and games (i.e., the program developed over forty modified formats) and dedicated education and mentoring, it also directly supported the professional development of community level coaches (including volunteer parents) and teachers across the national network of primary schools to bolster the foundational levels of sport (24, 46). The contribution and legacy of this ground-breaking program to Australian sport at all levels, cannot be understated.

### Change in national system and impact on talent promotion

Since late 2012 and the advent of the federal government's *Winning Edge* policy in response to Australia's poor performance at the London Olympics (47), national talent identification within Australia is best described as *transitional* (29) and has occurred in direct response to a change in role of the AIS, rather than a transformational progression within the discipline. Apart from the short-lived *AIS Sports Draft* (2013–2016), national-level identification and development has been the responsibility of NSOs and their partner SSOs and SIS/SAS to implement within their respective systems. For instance, the state government of Queensland recently increased their investment into the Queensland Academy of Sport, to drive and manage state-based talent identification and development programs (including several sport science/medicine and coaching roles) in the lead-up to the 2032 Brisbane Olympics through its *Youfor2032* initiative launched in early 2022 by its Chief Executive and former UK Sport director, Chelsea Warr (48). Chelsea's contribution to the growth of talent promotion within the United Kingdom will be addressed in the next section.

Another major change bestowed by the *Winning Edge* policy, was the decision that the AIS would no longer deliver and manage high-performance programs and provide athlete scholarships, and funding would instead be “put back in the hands” of prioritised NSOs to administer and manage. Furthermore, in May 2018, the dedicated sports science and medicine workforce located within the AIS hub in Canberra was significantly reduced, leading to the firm contention, that the AIS no longer resembled the vibrant and world leading institution, envisioned and realised through the 80s, 90s and 2000s (49, 50). As renowned AIS historian and scholar Greg Blood (51) shared, “*It (AIS) has now changed from an elite sport training centre with the mantra of ‘athlete- centred, coach-driven’ to a centre where sports and their coaches and athletes are clients or customers to AIS facilities and services.*” The current AIS model which garners substantial federal funding despite not delivering sport programs and offering athlete scholarships, continues to administer federal funding and hosts “user-pay” camps for NSOs, features a small cohort of “national discipline leads” specific to each sport science/sports medicine discipline who provide guidance across the broader network of providers within NSOs and the SIS/SAS, and provides guidance and grants specific to high performance coaching, performance pathways and athlete wellbeing and engagement which is limited to recognised sports and nationally categorised athletes, and not below (52).

High-performance funding for recognised Olympic NSOs overseen by the AIS, is over a four-year span in accordance with the Olympic cycle and the collective investment is smaller in magnitude than the United Kingdom, as will be discussed later. NSOs are required to submit annual plans to the AIS and report on their achievement of key performance indicators specific to their high-performance operations only (e.g., international level performances) and not inclusive of their underpinning but critically important talent pathways and operations. To support the achievement of these short-term high-performance targets and ensure ongoing federal funding, the predominant spend of NSOs are within the high-performance levels inclusive of its *performance pathways* that supports nationally categorised athletes only, and not supporting sustainable talent promotion of emerging athletes below a nationally categorised level.

**Figure 5:**
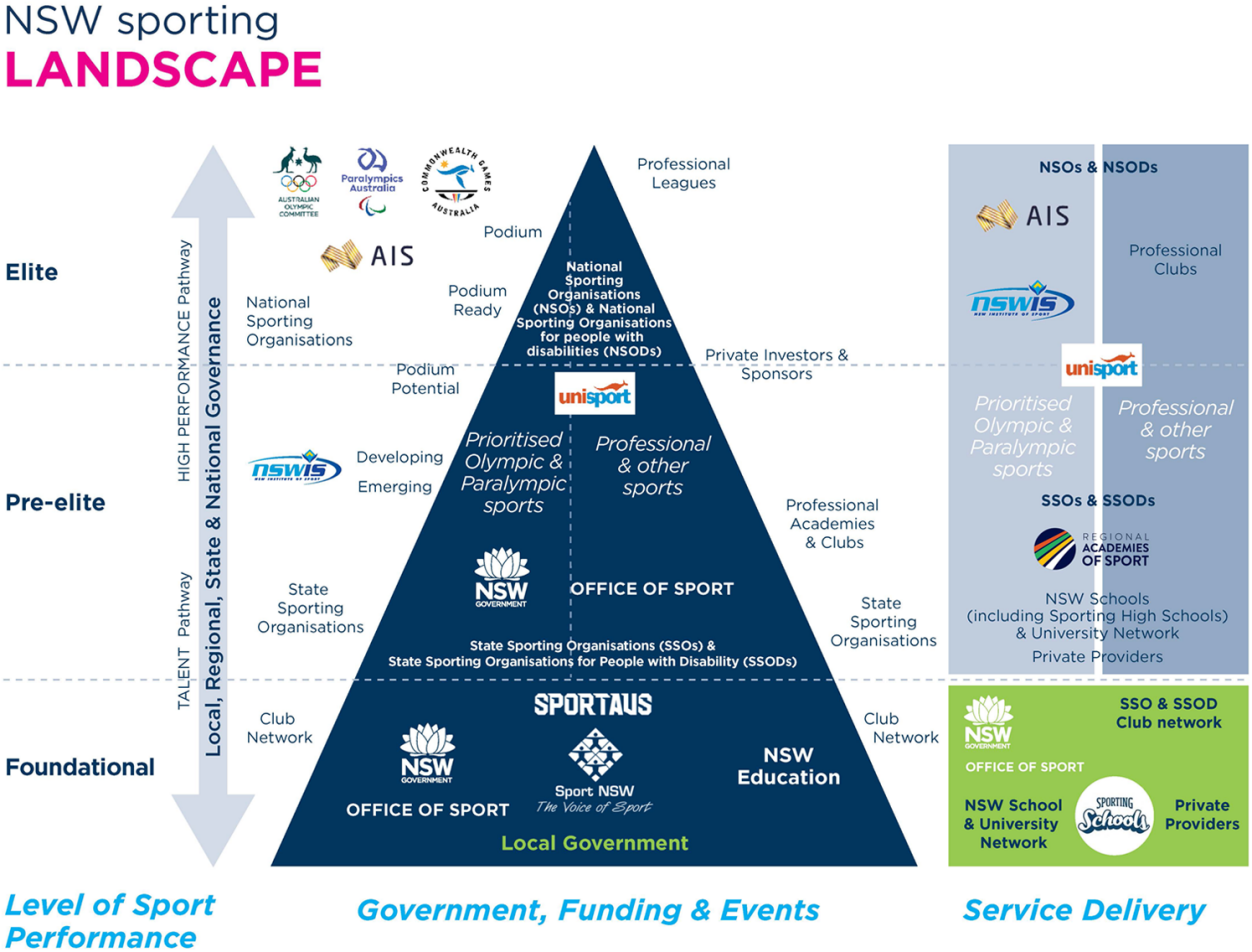
Sporting sector landscape of New South Wales (53). Image reproduced courtesy of New South Wales Office of Sport.

Additionally, NSO's are required to align and coordinate several underpinning state and territory, regional and local level organisational partners and across the sport continuum from early participation to high performance [see Figure 5 specific to the sporting landscape within the state of New South Wales]. Without an effective and evolving “whole of sport” strategy and the compliance, alignment, and collaboration of system stakeholders, it can be challenging and inefficient. Since the advent of the *Winning Edge* policy, the *FTEM* (Foundation, Talent, Elite and Mastery) athlete development framework (41, 54) developed within the AIS and operationalised through the 3D-AD (Three Dimensional Athlete Development) model [see Gulbin and Weissensteiner (55) and Weissensteiner (54, 56) for more information], has been utilised extensively by many national, state, and regional sporting organisations, to inform the review and refinement of their “whole of sport” planning, implementation and evaluation inclusive of talent promotion [see Weissensteiner (54),]. A notable adopter of this approach is Swimming Australia. Since 2014, Swimming Australia has implemented and evolved the *Australian Swimming Framework* (ASF) to support its operational alignment, effectiveness and success (57). At the recent 2021 Tokyo Olympics Australian swimmers won nine gold medals, more than half of Australia's overall tally of seventeen (58). Whilst Swimming Australia amongst other NSOs have embraced and committed to this “whole of sport” planning approach, it is not a mandated requirement by the AIS, nor Sport Australia for recognised sports.

At a federal policy level, Australia has lacked for some time a dedicated national coaching strategy spanning the *trichotomy* of sport (i.e., participation, talent, and high performance). Like the current federal sport strategy *Sport 2030: Participation, Performance, Integrity, Industry* (59) and funding of NSOs, there has remained for some time, an uncomfortable juxtaposition between supporting the community base of coaching and high-performance, with coaches within the critical talent pathways lacking advocacy, funding, resources, and support. The ASC has recently re-invigorated its approach to educating and supporting community level coaches which shows great promise. The AIS currently provides grants to NSOs to support the acquisition of coaches within its performance pathway and high-performance levels and grants to support coach professional development, but this is limited to coaches of nationally categorised athletes and not below that. Coaches within the critical talent levels operating at a local, regional, and state level are commonly poorly renumerated or volunteer, work mostly on a part-time or casual capacity, and have limited access to ongoing professional development. Within the state of New South Wales and guided by *the FTEM NSW Participant and Athlete Development Framework* (60, 61), the NSW Office of Sport released its *Future Champions* strategy in December 2019 (53) and in early 2022 it's *Phase One Action Plan* (62) - the first state-level, systems strategy dedicated to building and sustaining the foundational and talent pathways of sports. A key priority of this initiative alongside facilitating system leadership and a best practice approach to talent promotion is boosting the capability and capacity of coaching talent within NSW.

### Closing comments

With Australia's next home Olympics, Brisbane 2032 on the horizon, enthusiastic discussion specific to revisiting its national approach to talent promotion has been re-invigorated. Former Chief Executive of the Australian Sports Commission and respected advocate Jim Ferguson, contends that the ASC and the AIS must return to the roles defined for them in the Australian Sports Commission Act 1989 and that NSOs be supported in developing and implementing “whole of sport” plans and strategies inclusive of best practice talent promotion [see Ferguson (63, 64)]. Further discussion specific to the viability of National Lottery funding (much like that of the United Kingdom) to further invest into Olympic talent and performance pathways may be reinvigorated (65, 66).

A keen observer (amongst many) of Australia's journey has been the United Kingdom. In the next section, we chronicle the evolution of its national talent promotion system and its linkage with the former systems.

## United Kingdom

Empowered through strong leadership, shared vision, sustainable investment, effective coordination and firm governance and benefitting from learnings stemming from the Australian system (which was in turn influenced by the former German Democratic Republic), the United Kingdom possesses arguably one of the most advanced and successful talent promotion systems in the world, contributing to its perennial high-performance success over the last four Olympic cycles (see Figure 1). As Dennis and Crix (2) observed, “*While no commentators would agree that the contemporary UK elite sport system is based upon or moving towards a version of the East German one, many would concede that the UK looked for inspiration to the successful Australian system, which was itself modelled to a great extent on the GDR template. Thus, we have the ‘transfer’ of ideas, techniques, and structures – such as the need for a systematic talent identification programme – that derive from the GDR, are then interpreted and implemented in Australia, and later influence and are incorporated into the UK's understanding of what it takes to achieve elite success*” (p. 175).

In the following section, we chronicle the evolution and highlight the core components of this leading national talent promotion system.

### Historical background and genesis

Like Australia, the impetus for change and the need for direct national governmental intervention to systemise talent promotion in the United Kingdom, was declining Olympic level performance. At the 1996 Atlanta Olympics, the United Kingdom won a solitary gold medal and placed 36th on the medal table. In direct response, then Prime Minister, John Major oversaw a substantial review and restructure of the sports system including the establishment of its high-performance agency, UK Sport, in January 1997 and home nation sport councils.

Further justification for change, came in July 2005 when the United Kingdom was successful in its bid to host the 2012 London Olympics. In 2006, UK Sport under the leadership of its inaugural chair, Sue Campbell established and committed to operating and mandating its *World Class Performance Pathway* inclusive of progressive levels, *World-Class Talent* (i.e., athletes considered to be eight years away from reaching podium)*, World-Class Development* (i.e., athletes four to six years away from podium) and *World-Class Podium* (i.e., athletes four years and less from podium) and a “*no-compromise*” approach to funding its numerous Olympic and Paralympic National Governing Bodies (NGBs) contingent upon strict planning and review requirements, results from the prior Olympics, its competitive track record, projected medal capability and demonstrated ability to produce athletes through the pathway inclusive of the talent levels, articulated more recently within its *Code for Sports Governance* (67). Sue Campbell proclaimed, “*… UK Sport will take full responsibility for identifying and then supporting our most talented athletes, streamlining the system, and giving all Olympic and Paralympic sports a ‘single front door’ for funding and support. In a devolved world that is as close to the single agency model as you are ever going to get*” (68).

It is pertinent to note that concurrent to her role as chair of UK Sport, Sue Campbell, a former teacher who was equally passionate about the role that schools play in facilitating physical literacy and its contribution to “academic literacy”, was the inaugural Chief Executive of the Youth Sport Trust (YST), a charity championing youth engagement in physical education and sport in schools and clubs. Like its Australian predecessor *Aussie Sport*, the program featured a dedicated national physical education curriculum implemented across the national school network, provided ongoing professional development opportunities for teachers and coaches, and established an athlete leadership program *Our Changing Lives* (69). Additionally, the YST developed and implemented the *National Physical Literacy Framework* and award-winning *Girls Active campaign* in 2014 and continues to host the *UK School Games* a four-day multi-sport national event for emerging school-aged athletes (69).

Since 2006, UK Sport has served as the leading high-performance agency in the United Kingdom, providing centralised strategic support on behalf of the system, oversees the establishment and periodic review of an NGB's “*whole of sport*” operational plan inclusive of its talent strategy, development of world-class coaches and pathway managers and delivery of targeted talent identification and development campaigns, all of which will be discussed in detail later in this section. It's “*no compromise approach*” received criticism domestically, with critics saying it (the United Kingdom system) had “…*gone too far and (was) damaging grassroots sport*” particularly in sports such as basketball that historically received less funding and support (70).

Affiliates of UK Sport from within each of the four home nations include Sport England, Sport Scotland, Sport Wales and Sport Northern Ireland all of which support grass roots participation and community sport but also talent pathways and high-performance through their respective national centres of excellence (e.g., English Institute of Sport, Scottish Institute of Sport, Northern Irish Institute of Sport and the Welsh Institute of Sport) who in turn, support a network of underpinning regionally based institutes or academies. UK Sport and each of these home country agencies are entrusted with managing the United Kingdom's governmental investment into the sport system sourced from its exchequer (tax) and the National Lottery (71). This ongoing investment funds the operations of UK Sport including its coaching and performance pathways initiatives, is administered as grants for recognised NGBs and payments to athletes including the Athlete Personal Award (APA) and supports multi-disciplinary sport science/medicine and performance lifestyle support for athletes.

Funding to prioritised NGBs is spread over the four-year Olympic cycle but within the context of a twelve-year projection to support long-term system sustainability and growth. Unlike the Australian system, the allocated investment into the talent and performance pathways levels of Olympic NGB's is effectively “ring fenced” - dedicated solely to supporting this critical and recognised component of the high-performance system.

In establishing its system, UK Sport fervently recruited “expertise” - administrators, sports scientists, and coaches from across the globe, including from Australia. Notable appointments included Wilma Shakespear, former head coach of the AIS netball program and director of the Queensland Academy of Sport who became the inaugural director of the English Institute of Sport, her husband Peter Shakespear recognised earlier, who established British Rowing's highly successful *World Class Start* talent identification and development program, former AIS head swimming coach Bill Sweetenham who became national performance director of British Swimming (2000–2007), his successor at British Swimming (2007–2013) former AIS director (2001–2005) and inaugural director of the NSW Institute of Sport, Michael Scott, talent practitioner Chelsea Warr a physiologist who formerly worked within Australia's National Talent Search program [see Hoare and Warr (72)] and rowing coach Paul Thompson recognised earlier within this chapter.

The national sport agency of each home nation in partnership with its high-performance institute, oversee the implementation of their respective talent pathways plans (*Performance Foundations*) which underpins and contributes directly to UK Sport's *Performance Pathway* (see Sport England's 2018 released *Talent Plan for England – Creating the world's best talent system* as an example).

Unlike the Australian system, the investment into each home country's talent system and plan is substantial. For instance, within Sport England's Talent Plan (73), £85 million pounds were invested into its talent system (2017–2021 funding cycle) which directly supported England Talent Pathways (ETPs) that contribute to both national and commonwealth high-performance outcomes, within forty-three sports. The scale and breadth of operations is substantial - supporting approximately 60,000 athletes directly within ETP programmes and 200,000 emerging youth athletes within its underpinning entry level talent programs who receive coaching and competition support (73). Athletes supported through the ETP, are eligible for the Talented Athlete Scholarship Scheme (TASS), a Sport England-funded partnership between NGBs and educational institutions to provide dual career support.

Supporting the talent pathway within each home nation, are their network of schools (including a small number of specialised sports schools), colleges and universities which provide athletes with valuable participation and competitive opportunities within their respective sports clubs and teams and access to facilities and coaching. Additionally, each NGB has a network of affiliated clubs that “…*provide and facilitate the ‘daily training’ facilities, camaraderie, coaching and governance structures necessary to support talented athletes*” (73).

### Consolidated talent identification and development strategy

After working within British Swimming as a Talent Identification manager, Australian Chelsea Warr joined UK Sport in 2005 and led the formulation of its *Talent Identification and Performance Pathways* section, later becoming Director of Performance. Through Chelsea's leadership and exploration of expertise within other “performance” domains such as medicine, the team established and mandated across the system, a methodical multi-staged process for supporting effective talent identification and development (comparable to that advocated through the talent levels of Australia's FTEM Athlete Development Framework).

Following successful submission of their application, athletes were required to attend one of many dedicated testing centres and undergo two phases of *talent identification.* The first phase involved anthropometric and physiological testing and consideration of an athlete's training and competitive history. The second phase, involved follow-up sport-specific testing to ascertain an athlete's sport suitability, undergo a functional movement screening, and psychological and behavioural assessments. The successful athlete was then required to go through a dedicated *confirmation* phase whereby they were formally inducted and embedded within a dedicated daily training environment for their sport for 6–12 months to verify their readiness, commitment, and developmental and performance potential. Progressing from this phase, emerging athletes were supported through a longer *development* phase in which they received individualised athlete planning, access to quality coaching and core sport science/medicine services, athlete education, career mentoring and “*performance lifestyle*” support, and access to progressive competition.

Adopting this approach since 2007, UK Sport has successfully delivered seventeen talent initiatives featuring both traditional (i.e., selection of existing talent within a sport) and non-traditional talent recruitment approaches (i.e., talent detection and transfer).

Following Australia's lead, talent transfer or “reassignment” of high-performance athletes into another sport after exiting their prior sport, has proved to be a very fruitful strategy that has translated into “fast tracked” and substantial Olympic success for the United Kingdom. The *Girls4Gold* program was UK Sport's inaugural talent transfer program launched in 2008, whereby British female athletes aged between 17 and 24 years of age, who possessed the attributes of power, strength, speed, and mental toughness, were recruited. Successful athletes were then embedded into well resourced, dedicated developmental programs within the sports of skeleton, canoeing, modern pentathlon, rowing, and sailing. Several Olympic champions have been unearthed through this approach including two-time gold medallist rower Helen Glover who was a former national level athletics representative and hockey player, and fellow rower and former equestrian showjumper Victoria Thornley, who competed at the 2012 London Olympics five years after she was talent identified and won a silver medal at the 2016 Rio de Janeiro Olympics (74). Another notable athlete discovered through the *Girls4Gold* program was former heptathlete Lizzy Yarnold, who became the United Kingdom's most successful Winter Olympian in the sliding sport of skeleton. Lizzy commenced competition in skeleton in 2010, became Junior World Champion in 2013 and then won back-to-back gold medals at the 2014 Sochi and 2018 PyeongChang Winter Olympics (75).

### Dedicated tools and ongoing review

UK Sport's Performance Pathways personnel established the dedicated benchmarking tool and evaluative process, known as the *Pathway Health Check*. This tool administered every four years, serves to benchmark against “world's best,” identify gaps and opportunities within the sport, which in turn, facilitate discussion and the workshopping of viable solutions with NGB Pathway staff. The focus areas of the tool include “*a gap analysis, athlete profiling, junior to senior transition, retention/attrition rates of athletes in the pathway, confirmation processes and the effectiveness of the development curriculum the athlete receives*” (76). As well as guiding an NGB's pathways strategy and operations, it also provides critical intelligence of the sector for UK Sport to further inform and refine their overarching high-performance strategy and prioritisation. The AIS through its former Athlete Pathways and Development section, developed a similar tool in 2013, the *NSO Pathway Healthcheck* to support the review and refinement of an NSO's pathway strategy and implementation [see Weissensteiner (54)]. A point of difference, however, is that unlike the United Kingdom's tool, there are no funding implications for Australian NSOs.

### Dedicated ongoing investment into talent promotion workforce

Supporting the effective implementation, alignment and growth of UK Sport's talent promotion strategy is its ongoing investment into its dedicated “talent workforce” (i.e., NGB pathway managers and coaches) and the fruitful ongoing partnership with its university sector. For many years, UK Sport has directly supported the professional development and support of NGB pathways managers through its dedicated talent curriculum (e.g., *World Class Talent, Confirmation and Development – A framework for talent managers and coaches*), educational and networking opportunities such as pathway symposiums and masterclasses, to instil a best-practice and progressive approach to talent promotion and grow the capability of its workforce. More recently, UK Sport has established an online *Performance Pathways Learning Hub* to support ongoing education.

Coaching is recognised as a central pillar of the United Kingdom's talent promotion system. In 2008, the *UK Coaching Framework* was launched to support an increase in the capability and capacity of coaches at all levels of sport. Sue Campbell declared at its inception in 2008, that “…*its implementation will raise the standard and sustainability of coaching in the UK, promoting a clear pathway for the development of world-class coaching expertise from grassroots to elite level”* (77). Since this time, substantial, ongoing investment into the professional development of its coaching workforce inclusive of those within the talent and performance pathways in its progressive *Foundation, Apprenticeship* and *Elite* programs, has ensued [see UK Sport (77)]. All coaches aligned and supported through the strategy receive individualised education and development, on the job training and feedback, ongoing mentoring, and access to periodic networking opportunities such as conferences and events (77). Concurrently, *UK Coaching*, an active charity which currently supports three million coaches across the United Kingdom, provides best practice education and training, aligned research, and maintains industry standards across sports, communities and NGBs (78). The *Coach Learning Framework* is an exemplary ecological and practical tool developed by UK Coaching to directly support coaching capability and includes advice specific to athlete development but also self -reflective practices, lifestyle, and wellbeing tips (79).

Extending upon the engagement with the university sector that featured within Australia's former National Talent Identification and Development program's “*talent assessment centres*”, the United Kingdom's talent promotion system features strong linkage and expansive contribution from its university sector including access to quality sporting infrastructure, training facilities and equipment, athlete testing and personnel, ongoing research and innovation and delivery of core sport science/sports medicine services. The EIS high-performance centre at Loughborough University for instance, supports athletes from a wide range of sports and provides sports science/sports medicine services across the East Midlands of Great Britain in partnership with the Holme Pierrepont Sports Centre in Nottingham (80). Similarly, the EIS high-performance centre based at the University of Bath is the training base for several sports including modern pentathlon, bobsleigh, skeleton, and swimming and supports the delivery of sport science/sports medicine services across the southwest of Great Britain complementing services provided in Weymouth (sailing) and Plymouth (diving) (80).

### Research and innovation informing strategy and practice

UK Sport has invested substantially into ongoing research and innovation to enhance its approach and delivery of talent promotion. For example, the *Great British Medallists* research project, commissioned by UK Sport and led by Bangor University's Institute for the Psychology of Elite Performance, was implemented to gain an evidence-based understanding of world class athlete development by exploring the developmental histories of thirty-two former British Olympic athletes, half of which were categorised as “*super elite*” (i.e., won an Olympic or World Championship gold medal and another medal at that level) and the other half, “*elite*” athletes (i.e., had not won a medal at that level but were recognised and supported high performance athletes) [see Rees et al. (81)]. Insights garnered from the project further informed UK Sport's pathway strategies and implementation including the professional development of its coaches, performance directors, pathway managers, and other officials supported through the World Class Programme.

### Final comments

Capitalising on the keen observations and learnings from the former German Democratic Republic and Australian systems and unashamedly reliant upon substantial ongoing federal government investment, the United Kingdom through its enduring and effective leadership and structures, firm governance, dedicated high performance plans inclusive of the underpinning talent pathways and coordinated and collaborative capable network, has developed a world-class system of sustainable talent promotion, admired across the globe.

## Discussion – looking to the future

In this chapter we explored the growth of national level talent promotion by chronicling the emergence and contributions of the former German Democratic Republic, Australia and the United Kingdom. As our exploration revealed, despite their apparent differences in political ideology, intent and ethics, there were key strategic and operational similarities and linkage between the systems. Whilst it is quite evident that this transference or “mirroring” of policy and operational elements has occurred between these national systems, successful adaptation and implementation within the “recipient” country is contingent upon and enabled through, the “right fit” of leadership (and courage!), expertise and innovation. As Gulbin (29) states, “…*international sporting systems are becoming more uniform than different. This suggests that there are few secrets in elite sport, but rather the point of differentiation being a nation's ability to optimally coordinate these common components*” (p. 147).

Declining physical literacy and youth sport participation incurred by the Covid-19 global pandemic (82) present ongoing challenges for current and future talent promotion, that must be recognised, and future policy and implementation must consequently be adaptive.

In addition to analyses of efficiency and cost-benefit at a national level [see Güllich & Emrich (4, 14)], it is strongly advocated that future researchers adopt ethnographic, ecological, and transdisciplinary approaches [see Toohey et al. (83)] to evaluate national-level talent promotion from a systems-management perspective (i.e., in their operational entirety inclusive of each level and relative integration) and not compartmentalised (i.e., focussed on one discrete aspect within one level).

**Figure 6:**
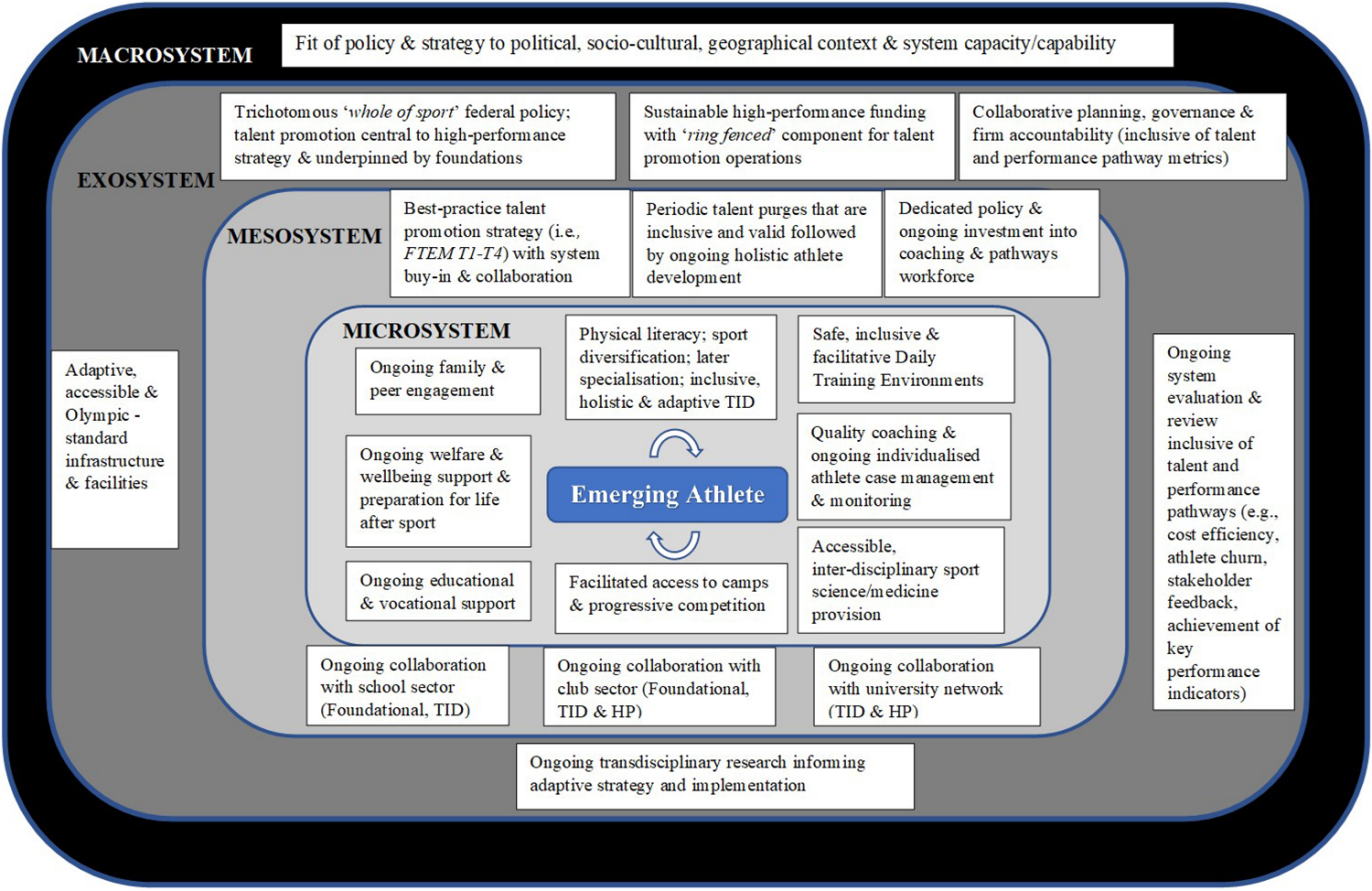
System level and integrated framework to support effective national talent promotion.

Whilst this chapter has focussed on well-resourced nations characterised by substantial capacity, we contend that its key learnings, offer good guidance for any policy makers and practitioners assisting with the review and refinement of their respective national systems. Based on our collective learnings of the prior system and considerate of contemporary issues, we provide in Figure 6 for consideration, a systems framework outlining key aspects (i.e., strategic, operational etc.) at a macro, exo, meso and micro level to support effective and sustainable national-level talent promotion.
